# The Effects of Incremental Doses of Aflatoxin B_1_ on In Vitro Ruminal Nutrient Digestibility and Fermentation Profile of a Lactating Dairy Cow Diet in a Dual-Flow Continuous Culture System

**DOI:** 10.3390/toxins15020090

**Published:** 2023-01-18

**Authors:** Felipe Xavier Amaro, Yun Jiang, Kathy Arriola, Matheus R. Pupo, Bruna C. Agustinho, Sarah L. Bennett, James R. Vinyard, Lais Tomaz, Richard R. Lobo, Andres Pech-Cervantes, Jose A. Arce-Cordero, Antonio P. Faciola, Adegbola Tolulope Adesogan, Diwakar Vyas

**Affiliations:** 1 Department of Animal Sciences, University of Florida, Gainesville, FL 32611, USA; 2 College of Agriculture, Community and the Sciences, Kentucky State University, Frankfort, KY 40601, USA; 3 Department of Animal and Dairy Sciences, University of Wisconsin-Madison, Madison, WI 53706, USA; 4 Department of Animal, Veterinary and Food Sciences, University of Idaho, Moscow, ID 83844, USA; 5 Department of Animal Science, Penn State University, University Park, PA 16803, USA; 6 Department of Animal Breeding and Nutrition, Sao Paulo State University, Botucatu 18610-307, SP, Brazil; 7 Agricultural Research Station, Fort Valley State University, Fort Valley, GA 31030, USA; 8 Escuela de Zootecnia, Universidad de Costa Rica, San Jose 11501-2060, Costa Rica

**Keywords:** aflatoxin B_1_, mycotoxin, nutrient digestibility, N metabolism, rumen fermentation, ruminal microbial protein

## Abstract

Aflatoxin B_1_ (AFB_1_) is a mycotoxin known to impair human and animal health. It is also believed to have a deleterious effect on ruminal nutrient digestibility under in vitro batch culture systems. The objective of this study was to evaluate the effects of increasing the dose of AFB_1_ on ruminal dry matter and nutrient digestibility, fermentation profile, and N flows using a dual-flow continuous culture system fed a diet formulated for lactating dairy cows. Eight fermenter vessels were used in a replicated 4 × 4 Latin square design with 10 d periods (7 d adaptation and 3 d sample collection). Treatments were randomly applied to fermenters on diet DM basis: (1) 0 μg of AFB_1_/kg of DM (Control); (2) 50 μg of AFB_1_/kg of DM (AF50); (3) 100 μg of AFB_1_/kg of DM (AF100); and (4) 150 μg of AFB_1_/kg of DM (AF150). Treatments did not affect nutrient digestibility, fermentation, and N flows. Aflatoxin B_1_ concentration in ruminal fluid increased with dose but decreased to undetectable levels after 4 h post-dosing. In conclusion, adding incremental doses of AFB_1_ did not affect ruminal fermentation, digestibility of nutrients, and N flows in a dual-flow continuous culture system fed diets formulated for lactating dairy cows.

## 1. Introduction

Aflatoxins (AF), produced by *Aspergillus* spp., such as *A. parasiticus* and *A. flavus* [1], are fungal metabolites that can be found in several foods and feed [2]. There are six known forms of AF: AFB_1_, AFB_2_, AFG_1_, AFG_2_, AFM_1_,and AFM_2_; the first four are predominantly found in plant source foods and feed, whereas the other two are found in animal source foods, such as dairy products from cows fed contaminated feed [2,3]. Because of their mutagenic, teratogenic, and carcinogenic effects under long-term exposure [4], AF are amongst the most dangerous compounds capable of altering physiological processes in animals and humans even when present in trace amounts [5,6].

The US Food and Drug Administration has established 20 μg/kg as the action level for AFB_1_-contaminated feeds destined for dairy cows. Aflatoxin B_1_ can be transferred from feed to milk as AFM_1_ and action levels (limit) of 0.50 μg/kg of milk have been established for milk destined for human consumption [7]. In silages, AF contamination can occur during pre- and post-harvest processes but is mainly associated with poor storage [8,9]. Environmental factors, such as droughts, can favor AF synthesis in both silages and grains [9]. Similarly, poorly stored cereal grains provide favorable conditions, such as humidity and high temperatures, for the development of AF producing fungi [10,11,12].

The detrimental effects of AFB_1_, such as decreased ruminal nutrient digestibility, have been reported in vitro when AFB_1_ was examined at concentrations greater than 200,000 μg/kg [13] and 640 to 1920 μg/kg [14]. Based on a worldwide 3 yr (2009–2011) survey, 33% of the 4627 feed samples analyzed were AF positive, and the average concentration for contaminated feeds (corn, soybean meal, dried distillers grains with solubles, and finished feeds) was 63 μg/kg [15]. Hence, high doses of AFB_1_ used in previous in vitro studies may not reflect the concentrations of naturally occurring contaminated feeds. Furthermore, when dairy cows were challenged with dietary 100 μg of AFB_1_/kg of diet DM, the concentration in ruminal fluid was 0.20 μg/kg and milk AFM_1_ was increased to 0.80 μg/kg of milk, which was above FDA action levels [16]. This means that physiological detoxifying AFB_1_ mechanisms can decrease AFB_1_ concentration in the organism but are not effective in decreasing AFM_1_ in milk to concentrations below the FDA action level. Indeed, previous research has demonstrated the efficacy of several plant metabolites, such as curcumin, epigallocatechin gallate, and glutathione, at alleviating the oxidative stress caused by the circulating AFB_1_ and decreasing damage to organ tissues, such as the liver and kidney [17,18].

To the best of our knowledge, only one recent study examined the effects of a close to naturally occurring concentration of AFB_1_ (75 μg/kg of feed DM) on ruminal digestibility using an in vitro batch culture system [19]. These authors reported decreased dry matter (DM) digestibility and increased NH_3_-N and acetate concentration for aflatoxin-inoculated diets compared to the aflatoxin-free control; however, AFB_1_ recovery under ruminal conditions was not evaluated. Furthermore, a recent review of the literature concluded that mammals and humans lack strong intrinsic ruminal and cellular AF degradation mechanisms and that strategies, such as the use of yeast and bacteria products, should be employed to mitigate the adverse effects of aflatoxin-contaminated feeds [5]. Conversely, there is still disagreement on whether these technologies are effective [20]. Therefore, understanding the effects and dynamics of naturally occurring concentrations of AFB_1_ under ruminal conditions may shed light on the mechanisms of AFB_1_ clearance from the rumen and aid the development of strategies to mitigate the harmful effects of AFB_1_ on animals and humans. Our objectives were to assess the effects of incremental doses of AFB_1_ on ruminal fermentation, DM and nutrient digestibility, and N metabolism of a lactating dairy cow diet and to examine the AFB_1_ recovery in ruminal fluid using a dual-flow continuous culture system. We hypothesize that detrimental effects of AFB_1_ on ruminal nutrient digestibility will increase with an increasing dose of AFB_1_ and that a greater dose will promote greater AFB_1_ recovery in ruminal fluid.

## 2. Results

The basal diet provided 15.9% of crude protein (CP) and 1.61 Mcal/kg of DM (Table 1), and it was similar to the diet fed to the lactating ruminal content donor cows. Diet composition was similar across all the treatments, differing only in the dose of AFB_1_ applied to each fermenter.

Ruminal pH, NH_3_-N, and total volatile fatty acids (VFA) averaged 6.08, 10.22 mg/dL, and 139.1 m*M*, respectively, and were not affected by AFB_1_, regardless of the dose used (*p* > 0.10; Table 2). In addition, no linear, quadratic, and cubic effects of AFB_1_ dose on pH, NH_3_-N, and total VFA were detected (*p* > 0.10). Similarly, no AFB_1_ × sampling time interaction was detected for NH_3_-N, pH, lactate, and VFA (*p* > 0.10; Supplemental Figures S1–S10). Molar proportions (mol/100 mol) of acetate, propionate, butyrate, iso-butyrate, valerate, and iso-valerate averaged 50.92, 28.11, 12.98, 0.50, 4.75, and 2.67 mol/100 mol, respectively, while lactate and acetate to propionate ratio (A:P) averaged 0.23 m*M* and 1.85, respectively. Individual VFA and ruminal lactate concentrations were not affected by AFB_1_, regardless of the dose used.

The dose of AFB_1_ did not affect true digestibility of DM, organic matter (OM), and CP and averaged 55.2, 62.2, and 60.1%, respectively (Table 3). Likewise, the dose of AFB_1_ did not affect apparent digestibility of neutral detergent fiber (NDF), acid detergent fiber (ADF), and starch (*p* > 0.10) and averaged 56.7, 41.1, and 93.0%, respectively. No linear, quadratic, and cubic effects of AFB_1_ were detected for digestibility.

Flows of total N, NH_3_-N, non-ammonia N (NAN), microbial N, and dietary N were not affected by AFB_1_. Similarly, ruminal microbial efficiency and N efficiency did not differ among treatments (Table 4) and no linear, quadratic, and cubic effects of AFB_1_ doses were detected. Flows of N averaged 2.34, 0.42, 1.91, 1.09, and 1.26 g/d for total N, NH_3_-N, NAN, microbial N, and dietary N, respectively, while ruminal microbial efficiency and N efficiency averaged 19.0 and 36.9%, respectively. Hence, ruminal N metabolism was not affected by the dose of AFB_1_.

**Figure 1:**
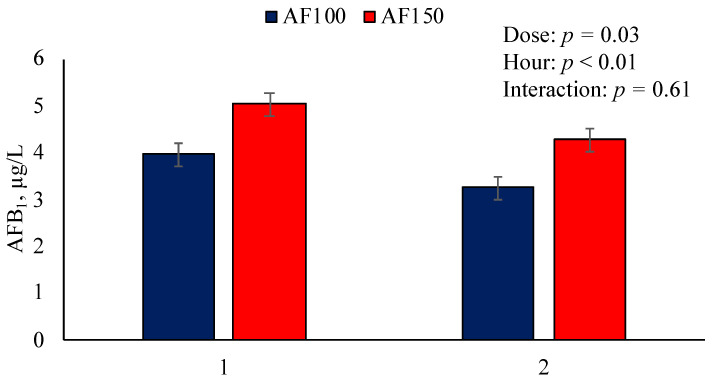
Effect of dose of aflatoxin B_1_ (AFB_1_) by sampling hour interaction on AFB_1_ concentration in ruminal fluid. After 4 h post-feeding, AFB_1_ was not detected in ruminal fluid using a commercial kit (AgraQuant^®^ Aflatoxin B_1_; Romer Labs); limit of detection of 2 μg/kg.

Aflatoxin B_1_ was not detected in Control and was not analyzed in AF50 samples because the final concentration in ruminal fluid was below the limit of detection of the kit used for AFB_1_ quantification. There was no interaction between AFB_1_ dose and sampling hour (*p* = 0.61). However, the highest dose increased ruminal AFB_1_ concentration (*p* = 0.03; Figure 1). Greater AFB_1_ concentrations were detected for AF150 (4.65 μg/L) compared with AF100 (3.61 μg/L), and AFB_1_ concentrations were greater 1 h post-dosing compared with 2 h post-dosing. Immediately before feeding and AFB_1_ dosing (0 h), AFB_1_ was not detected in the ruminal fluid on any of the treatments. Similarly, AFB_1_ was not detected 4 h post-dosing.

## 3. Discussion

Previous studies have investigated the effects of AFB_1_ on ruminal nutrient digestibility and fermentation using in vitro systems [13,14]. However, these studies tested doses that are over 30 times the naturally occurring average [15]. In the present study, we tested AFB_1_ concentrations that were no more than three times greater the average AFB_1_ concentrations observed in naturally contaminated feeds.

Previous in vitro studies have reported lower concentrations of VFA and NH_3_-N when AFB_1_ was dosed at either higher than naturally occurring (649–1920 μg/kg) [14] or naturally occurring concentrations (75 μg/kg) [19], suggesting deleterious effects of AFB_1_ on ruminal fermentation. Considering the effects of AFB_1_ on ruminal fermentation under in vitro conditions, the lack of effects on ruminal fermentation observed in this study were unexpected. However, these effects are in agreement with an in vivo study in which no changes on VFA concentrations were observed after AFB_1_ dosing in growing lambs [21]. We speculate that the lack of effects in the present study and under in vivo experimental conditions are due to the passage rate of AFB_1_ from the rumen resulting in rapid AFB_1_ clearance, thereby mitigating the detrimental effects of the aflatoxin on nutrient digestibility and fermentation.

The lack of treatment effects on fermentation probably reflects the lack of AFB_1_ effects on ruminal nutrient digestibility and N metabolism in the present study. Previous research reported a 50% decrease in the in vitro DM digestibility of alfalfa hay after 3 h of incubation with a dose of 2,000,000 μg of AFB_1_/kg of hay [13]. However, under a naturally occurring dose, a reduction of only 4% in the in vitro DM digestibility of a TMR was observed [19]. Aflatoxin B_1_-induced negative effects on nutrient digestibility may be due to reduced microbial activity and growth due to toxicity caused by AFB_1_ [13,19]. Several factors, including OM digestibility, affect ruminal microbial growth [22]. However, in the present study no treatment effects were observed on OM digestibility resulting in lack of effects on microbial N flow and microbial efficiency. Microbial efficiency of 17.5 g [23] and 18.4 g of microbial N flow/kg of digested OM [24] observed in previous studies using similar continuous culture fermenter system with comparable liquid and solid passage rates are in agreement with values observed in this study. In contrast, others reported 34.6 and 22.7 g of microbial N flow/kg of OM truly digested [25,26], respectively. In these studies, a buffer containing 0.1 g/L of urea was used, while the passage rate was 7 and 10%/h [25,26], respectively; however, in the present experiment, the buffer contained 0.4 g/L of urea and the system passage rate was 11%/h. Hence, variability in microbial efficiency between studies may be attributed to the differences in buffer solution and passage rates used.

Aflatoxin B_1_ recovery has been studied in vitro under ruminal conditions [14,27,28]. However, under in vivo conditions, AFB_1_ in the ruminal fluid has been quantified, but recovery over time has not been measured [16]. A study examining the recovery of six different mycotoxins, reported that AFB_1_ dosed at 200 μg/L of ruminal fluid had 100% recovery after 3 h of incubation, inferring no degradation of AFB_1_ [27]. In contrast, another study reported recoveries of 76 and 78% of AFB_1_ in ruminal fluid from lactating and dry cows, respectively, when 4.1 μg of AFB_1_/L of ruminal fluid was incubated for 1 h [28]. However, degradations of 83.1 and 84.2% of AFB_1_ after 72 h of ruminal incubation when AFB_1_ was dosed at 960 μg of AFB_1_/L on diets containing alfalfa hay or ryegrass hay, respectively, were reported [14]. Because dose and sampling time differed across these studies, an interaction between AFB_1_ dose and sampling time might have contributed to equivocal responses on aflatoxin degradation. For instance, at the higher dose used in some studies [14,27], ruminal fluid microbes might need over 3 h to degrade AFB_1_, while at the lower dose used by others [28], the clearance might have occurred within a couple of hours after dosing.

Previous studies examined the recovery of AFB_1_ using batch culture systems when AFB_1_ was dosed at concentrations greater than the naturally occurring average [14,27]. However, we used concentrations of AFB_1_ that are close to naturally occurring AFB_1_ concentrations in contaminated feeds. In addition, we used a dual-flow continuous culture system in this study that allowed sampling over time to test ruminal AFB_1_ recovery. Greater AFB_1_ ruminal concentration on AF150-treated samples confirm the efficacy of our treatments in increasing the concentration of AFB_1_ with increasing doses. Considering the average fermenter vessel capacity of 1.82 L and AFB_1_ doses of 50, 100, and 150 μg/kg DM, the concentrations of AFB_1_ in the ruminal fluid contents immediately after dosing should have been 1.47, 2.94, and 4.41 μg/L, for AF50, AF100, and AF150, respectively. However, based on the AFB_1_ concentrations observed 1 h post-dosing, the recovery rates were 134 and 114% for AF100 and AF150, respectively. Aflatoxin B_1_ was dosed twice daily (am and pm) and sampling for recovery estimation was conducted after the morning dosing. Recovery rates above 100% at 1 h post-dosing may imply the existence of residual AFB_1_ from the previous day; however, AFB_1_ was not detected at 0 h. Another explanation relies on the precision of the analytical kit used. If precision decreased with concentrations close to the limit of AFB_1_ detection, AFB_1_ concentrations at the lower dose analyzed might have been inflated. This is especially important considering that the recovery rate for AF100 was over 30% greater than what was applied. Additionally, the ruminal clearance rate of AFB_1_ between 1 and 2 h post-dosing was 18.2 and 15.2% (Supplemental Figure S11) for AF100 and AF150, respectively.

In a study where AFB_1_ was dosed to dairy cows at 100 μg of AFB_1_/kg of DMI, a recovery of 0.20 μg/kg of ruminal fluid was reported [16]. Because total ruminal contents were not measured in their study, it is challenging to calculate the recovery rate of AFB_1_ under in vivo conditions. Assuming an average DMI of 21.5 kg and rumen capacity of 120 L, 100% recovery of AFB_1_ in ruminal fluid would yield concentrations of 17.9 μg/kg. Nonetheless, only 1.12% of this value was recovered, implying degradation by ruminal microbes or clearance due to absorption or passage to the small intestine. Because of the high limit of AFB_1_ detection of the method used, we were not able to quantify the recovery of the mycotoxin 4 h post-dosing and consequently were not able to estimate the clearance kinetics of AFB_1_ in ruminal fluid. Hence, more research is needed to understand AFB_1_ clearance kinetics in ruminal fluid, and future studies aimed at determining AFB_1_ ruminal degradation should consider more sensitive methods [29] to detect AFB_1_ and better understand aflatoxin degradation kinetics.

## 4. Conclusions

Aflatoxin B_1_ dosed at 50, 100, and 150 μg/kg DM did not affect ruminal fermentation, digestibility of nutrients, and N flows in a dual-flow continuous culture system fed a lactating dairy cattle diet. Under our experimental conditions, AFB_1_ concentration in ruminal fluid increased with dose at 1 h but decreased to undetectable levels 4 h post-dosing. The AFB_1_ clearance in our model may be a function of microbial degradation, system passage rate, or the interaction of both factors. Further research using more sensitive methods of AFB_1_ detection is warranted to understand aflatoxin degradation kinetics under ruminal conditions.

## 5. Materials and Methods

The dairy cows used in this study for ruminal content collection were cared for in accordance with guidelines approved by the University of Florida Institutional Animal Care and Use Committee protocol number 202009849.

### 5.1. Experimental Design, Treatments, and Diet

Eight dual-flow continuous culture fermenters were used in a replicated 4 × 4 Latin square design. Each fermenter vessel was treated as an experimental unit. Four fermentation periods of 10 d each, consisting of 7 d of adaptation and 3 d of sampling were carried out. Fermenters were randomly assigned to 1 of 4 AFB_1_ doses on diet DM basis: (1) 0 μg of AFB_1_/kg of diet DM (Control); (2) 50 μg of AFB_1_/kg of diet DM (AF50); (3) 100 μg of AFB_1_/kg of diet DM (AF100); and (4) 150 μg of AFB_1_/kg of diet DM (AF150). Doses were added directly to fermenter vessels and corresponded to 0, 5.35, 10.7, and 16.05 µg of AFB_1_/d for Control, AF50, AF100, and AF150, respectively. A total of 5 milligrams of AFB_1_ powder (Sigma-Aldrich Co., St. Louis, MO, USA) were diluted in 5 mL of absolute ethanol as per the manufacturer’s recommendation. Subsequent dilutions were made to achieve treatment solutions containing 0, 10.7, 21.4, and 32.1 µg of AFB_1_/mL for Control, AF50, AF100, and AF150, respectively. According to each treatment, 250 µL of each respective solution was applied along with the feed into each fermenter vessel for the entire duration of the experimental period.

The basal diet was formulated to meet the nutrient requirements of a lactating Holstein dairy cow (680 kg of body weight) producing 42 kg of milk/d, 3.5% milk fat, 3% milk protein, and 4.8% lactose based on the NRC (2001) model. Corn silage was dried in a forced-air oven at 60 °C until the DM was 90% to allow for proper grinding of the feed. Dried corn silage, corn grain, soybean meal, and the mineral premix were ground through a 2 mm screen in a Wiley mill (A. H. Thomas Co., Philadelphia, PA, USA). Alfalfa hay and citrus pulp pellets were included as purchased in the diets. Each fermenter was fed 107 g of DM per day. The diet was fed in two equal allowances at 0800 (8 AM) and 1800 h (6 PM) daily. We chose two timings to mimic twice a day feeding of dairy cows.

### 5.2. Dual-Flow Continuous Culture System Operation and Experimental Period

A dual-flow continuous culture system [30] was used in the present study. Each fermenter vessel had an average 1.82 L capacity when filled to the point of the solid effluent outflow. Simulation of ruminal fermentation was achieved by continuous agitation (100 rpm), temperature (39 °C), and infusion of N_2_ gas, and artificial saliva solution. Nitrogen gas was infused at 200 mL/min to maintain an anaerobic environment. The artificial saliva [31] containing 0.40 g/L of urea was supplied at 3.05 mL/min to regulate liquid and solid passage rates of 11%/h and 5.5%/h, respectively. To check for system functionality, ruminal pH and temperature were measured twice daily immediately before the feed was delivered.

Two ruminally cannulated lactating Holstein cows were used as ruminal content donors. Donor cows were fed a total mixed ration containing (DM basis) corn silage (40%), alfalfa hay (3%), ground corn (27.3%), soybean meal 44% (15.5%), citrus pulp (9.2%), and mineral and vitamin premix (5%). Approximately 2 h after morning feeding, ruminal contents were manually collected and filtered through four-layer cheesecloth into prewarmed thermos flasks, which were kept airtight until transported to the laboratory for pooling across cows (50:50 mix; vol/vol). Pooled ruminal content was added to each prewarmed (39 °C) fermenter vessel until it reached the solid effluent outflow.

On d 5 of each period, artificial saliva was exchanged for ^15^N-enriched saliva containing 77 mg/L of labeled ammonium sulfate (Sigma-Aldrich Co.). To create a steady state of ^15^N, immediately before the artificial saliva was exchanged, a pulse dose of 173.3 mg of (^15^NH_4_)_2_SO_4_ 10.2% atom excess was added to each fermenter vessel. ^15^N-enriched saliva was used as a marker for the estimation of microbial protein synthesis. Background samples of artificial saliva and digesta (pooled liquid and solid effluent) were collected on d 5 before enriched saliva was used and kept at −20 °C until analyzed. From d 8 to 10 of each period, effluent containers, solid and liquid, were put in an ice bath at 1 °C to inhibit microbial fermentation and subjected to estimation of ruminal fermentation and nutrient digestibility. At the end of d 10, fermenters were disabled, disassembled, cleaned, and reassembled for the following period.

### 5.3. Fermentation Profile

Ruminal pH was measured in each fermenter vessel using a portable pH meter (Thermo Scientific Orion Star A121, Thermo Fisher Scientific Inc., Waltham, MA, USA) at 0, 1, 2, 4, 6, and 8 h post-morning feeding during d 8 and 9 of each period. Aliquots of approximately 15 mL of ruminal content from each fermenter were filtered through four layers of cheesecloth to obtain a 10-mL sample that was immediately acidified with 0.1 mL of 50% H_2_SO_4_ solution (*v*/*v*) and stored at −20 °C until further processing and analysis. Samples were thawed and centrifuged at 7000× *g* for 15 min at 4 °C. Approximately 2 mL of the supernatant was filtered through a 0.22 μm filter and analyzed for lactate and VFA using a Merck Hitachi Elite LaChrome HPLC system (L2400, Hitachi, Tokyo, Japan) and a Bio-Rad Aminex HPX-87H column (Bio-Rad Laboratories, Hercules, CA, USA). Briefly, the column was used in an isocratic elution containing 0.015 M H_2_SO_4_ in the mobile phase of HPLC with a UV detector (wavelength 210 nm; L2400, Hitachi) and a flow rate of 0.70 mL/min at 46 °C. The remaining supernatant sample was used for NH_3_-N concentration analysis [32] in a 96-well flat-bottom plate and phenol-hypochlorite solution. Additionally, digesta samples were analyzed for lactate, VFA, and NH_3_-N as earlier described. Digesta samples corresponded to solid and liquid effluents that were pooled after 24 h fermentation periods on d 8, 9, and 10. Effluent containers were weighed before the morning feeding and pooled using a hand mixer for 30 s; samples were kept frozen at −20 °C until further analyzed.

### 5.4. Nutrient Digestibility

Diet samples were ground through a 1 mm screen in a Wiley mill (A. H. Thomas Co.) and dried in an oven overnight at 105 °C for DM estimation. Samples were ashed at 550 °C for 5 h [33] for OM estimation. The concentration of N was determined by rapid combustion using a micro elemental N analyzer (Vario Micro Cube, Elementar Analysensysteme GmbH, Langenselbold, Germany) [34]. Crude protein concentration was calculated by multiplying N concentration by 6.25. Amylase-NDF and ADF concentrations were sequentially analyzed using a fiber analyzer (200/220, Ankom Technology, Macedon, NY, USA) [35]. For aNDF determination, sodium sulfite and heat-stable amylase (Type XI-A from *Bacillus subtilis*; Sigma-Aldrich Co.) were used. Ether extract was determined using a fat analyzer (XT20, Ankom Technology) [33]. Starch was analyzed using a colorimetric method [36].

For the estimation of ruminal nutrient digestibility, digesta samples collected on d 8, 9, and 10 were freeze-dried for DM determination and immediately ground using a mortar and pestle and analyzed for OM, N, aNDF, ADF, and starch as earlier described. To estimate ruminal true digestibility of nutrients, artificial saliva collected on d 5 was freeze-dried for DM estimation and analyzed for total N and ash as previously described. We used the following equation for nutrient digestibility estimation [37]:

Nutrient digestibility % (DM basis) = 100 × [grams of nutrient intake − (effluent grams of nutrient − saliva grams of nutrient − bacteria grams of nutrient)]/grams of nutrient intake.

### 5.5. Microbial Protein Synthesis and Ruminal N Metabolism

At the end of each experimental period, microbial pellets from each fermenter vessel were harvested [38]. Briefly, total fermenter contents were blended for 1 min and filtered through 4 layers of cheesecloth with 200 mL of saline solution (0.9% NaCl). To remove the remaining feed particles, the filtrate was centrifuged (Allegra X-15R Centrifuge, Beckman Coulter Life Sciences, Indianapolis, IN, USA) at 1000× *g* for 10 min at 4 °C. The supernatant was collected and centrifuged (Sorvall RC-5B Refrigerated Superspeed Centrifuge, DuPont Instruments) at 11,250× *g* for 20 min at 4 °C for isolation of the microbial pellet. The microbial pellet was resuspended in 200 mL of McDougall’s solution for pellet purification and centrifuged at 16,250× *g* for 20 min at 4 °C. The final microbial pellet was resuspended in distilled water and transferred to a new container and kept at −20 °C until further analysis. The microbial pellet was freeze-dried for DM determination and analyzed for ash, total N, and ^15^N abundance.

We used ^15^N as a marker for microbial protein synthesis, and it was analyzed on artificial saliva, background digesta (before ^15^N-enriched saliva was used), digesta, and bacteria samples according to the following procedure. Freeze-dried samples were processed in 2 mL microcentrifuge tubes using 2.0 mm zirconia beads and homogenized (Precellys 24, Bertin, Rockville, MD, USA) at 5500× *g* for 10 s. Samples were weighed in tin capsules using a microscale (Excellence Plus XP Micro Balance Mettler-Toledo GmbH, Laboratory & Weighing Technologies, Columbus, OH, USA), and 35 µL of K_2_CO_3_ solution (10 g/L) were added to alkalinize the samples; the suspension was dried overnight in a forced-air oven at 40 °C to volatilize ammonia [39]. Analysis of ^15^N was performed with a mass spectrometer (IsoPrime 100, IsoPrime, Naperville, IL, USA), and the results were obtained as the fractional abundance of isotopic fractions (^15^N/^14^N). The equations used for the calculation of ruminal N metabolism are described below:

Microbial N flow (g/d) = (NAN flow × % atom excess of ^15^N of NAN effluent)/(% atom excess of ^15^N of microbial pellet);

The percent excess of ^15^N of NAN (non-NH_3_-N) effluent was obtained by subtracting % atom ^15^N in the background from the % atom excess of ^15^N of NAN effluent [40].

NH_3_-N flow (g/d) = effluent NH_3_-N concentration (mg/dL)/1000 × [total effluent flow (g)/100];

NAN flow (g/d) = effluent grams of total N − effluent grams of NH_3_-N;

Flows of NH_3_-N, NAN, and N metabolism were determined [41].

Dietary N flow (g/d) = effluent grams of NAN − effluent grams of microbial N;

Microbial efficiency was determined as follows [40].

Microbial efficiency = grams of microbial N flow/grams of OM truly digested;

Efficiency of N used was determined as follows [41].

Efficiency of N use = (grams of microbial N/grams of available N) × 100;

### 5.6. Aflatoxin B_1_ Ruminal Recovery

Ruminal contents (5 mL) from each fermenter were collected at 0, 1, 2, 4, 6, and 8 h after the morning feeding on d 8 and 9. Samples were transferred to screw-capped tubes and frozen at −20 °C until analyzed. We used an ELISA-based kit (AgraQuant^®^ Aflatoxin B_1_, Romer Labs, Getzersdorf, Austria) to quantify AFB_1_ in the ruminal fluid. Briefly, samples were thawed, and 1 mL of ruminal content was mixed into 5 mL of 70% methanol (*v*/*v*), the mixture was allowed to shake for 3 min, and after settling, it was filtered (Serum Filter System; Fisher Scientific). After extraction, we followed the manufacturer’s instructions. Briefly, 200 μL of the conjugate solution and 100 μL of standard or samples were transferred into the dilution well and mixed. A total of 100 μL of the mixed solution was transferred to the antibody-coated wells and incubated for 10 min at room temperature. Contents were discarded, and wells were washed with deionized water five times and tapped to dry. Subsequently, 100 μL of the substrate solution was added to each antibody-coated well and incubated for 10 min at room temperature. Stop solution was added to each well, and the ELISA-plate was read at 450 nm using a microplate reader (Spectra Max 340PC, Molecular Devices Corporation, Silicon Valley, CA, USA).

### 5.7. Statistical Analysis

Data were analyzed using the GLIMMIX procedure of SAS (version 9.4; SAS Institute, Cary, NC, USA) as a replicated 4 × 4 Latin square design. Data were checked for normality using the UNIVARIATE procedure of SAS (version 9.4; SAS Institute) before analysis. Variables that were measured repeatedly over time (pH, lactate, VFA, NH_3_-N, and AFB_1_ ruminal fluid concentration) were analyzed according to Model 1:

Model-1: Y_ijklm_ = µ + D_i_ + T_j_ + (DT)_ij_ + F_k_ + P_l_ + S_m_ + E_ijklm_,

where Y is the dependent variable, D_i_ is the fixed effect of the ith dose (i = 1, 2, 3, 4); T_j_ is the fixed effect of jth sampling time (j = 1, 2, 3, 4, 5, 6); (DT)_ij_ is the interaction effect of the ith dose at the jth level; F_k_ is the random effect of the kth fermenter (k = 1, 2, 3, 4, 5, 6, 7, 8); P_l_ is the random effect of the lth period (l = 1, 2, 3, 4); S_m_ is the random effect of the mth square (m = 1, 2); and E_ijklm_ is the residual error. Errors within fermenters across sampling time, which are repeated measures due to sequential sampling, were modeled using the Akaike information criteria covariance structure (unstructured, compound symmetry, first-order autoregressive) with the lowest Bayesian information criterion. Variables, such as nutrient digestibility, N metabolism, and pooled lactate, VFA, and ammonia-N, were analyzed according to Model 2:

Model-2: Y_ijkl_ = µ + D_i_ + F_j_ + P_k_ + S_l_ + E_ijkl_,

where Y is the dependent variable, D_i_ is the fixed effect of the ith dose (i = 1, 2, 3, 4); F_j_ is the random effect of the jth fermenter (j = 1, 2, 3, 4, 5, 6, 7, 8); P_k_ is the random effect of the kth period (k = 1, 2, 3, 4); S_l_ is the random effect of the lth square (l = 1, 2); and E_ijklm_ is the residual error.

In addition, linear, quadratic, and cubic contrasts were tested to examine trends in effects of the dose. Significance was declared at *p* ≤ 0.05, and a tendency was declared at 0.05 < *p* ≤ 0.10.

## Figures and Tables

**Table 1:** Ingredient and chemical composition of the experimental diets.

Item	Experimental Diet
Ingredient, % of DM	
Corn silage	40.0
Alfalfa hay	20.0
Corn grain, ground shelled	20.0
Soybean meal, 44%	13.5
Citrus pulp	4.0
Mineral premix ^1^	2.5
Chemical composition, % of DM	
OM ^2^	93.3
CP	15.9
RDP ^2,3^	9.8
RUP ^2,3^	6.1
Andf ^2^	26.4
ADF ^2^	19.4
NFC ^2,3^	49.5
Starch	30.3
EE ^2^	2.6
NE_L_^2,3^, Mcal/kg of DM	1.61

^1^ Mineral premix containing 24.3% CP, 5.74% aNDF, 19.2% NFC, 0.98% EE, 50.9% ash; macromineral composition 8.6% Ca, 1.6% P, 0.29% K, 3.18% Mg, 10.06% Na, 3.63% Cl, 0.53% S; micromineral composition 67.3 ppm Co, 248 ppm Cu, 20.5 ppm Fe, 1340 ppm Mn, 0.42 ppm Mo, 1750 ppm Zn; ^2^ OM, RDP, RUP, aNDF, ADF, NFC, EE, NE_L_ = organic matter, rumen degraded protein, rumen undegraded protein, amylase-neutral detergent fiber, acid detergent fiber, non-fibrous carbohydrates, ether extract and net energy for lactation, respectively; ^3^ estimated using the NRC (2001) model.

**Table 2:** Effects of incremental doses of aflatoxin B_1_ (AFB_1_) on pH, NH_3_-N, VFA, and lactate pool (24 h) of a lactating dairy cow diet in a dual-flow continuous culture system.

Item	Treatment ^1^				SEM	*p*-Value ^2^		
	Control	AF50	AF100	AF150		L	Q	C
pH	6.08	6.04	6.11	6.10	0.06	0.65	0.97	0.83
NH_3_-N, mg/dL	10.3	10.1	9.56	10.9	1.02	0.66	0.47	0.35
Total VFA ^3^, m*M*	137.5	141.8	142.9	134.4	8.23	0.78	0.76	0.38
Molar proportion, mol/100 mol								
Acetate	52.0	49.7	50.0	51.9	1.90	0.62	0.91	0.28
Propionate	27.4	28.9	28.7	27.5	1.34	0.67	0.90	0.39
Butyrate	12.8	12.3	13.6	13.2	0.55	0.18	0.39	0.37
Iso-butyrate	0.48	0.45	0.54	0.54	0.06	0.21	0.72	0.33
Valerate	4.51	5.38	4.73	4.38	1.10	0.86	0.55	0.12
Iso-valerate	2.78	2.95	2.51	2.42	0.39	0.33	0.90	0.33
Lactate, m*M*	0.23	0.24	0.27	0.17	0.07	0.86	0.45	0.39
A:P ^4^	1.92	1.74	1.84	1.91	0.12	0.87	0.63	0.25

^1^ Control = 0 μg/kg, AF50 = 50 μg/kg, AF100 = 100 μg/kg, and AF150 = 150 μg/kg; ^2^ contrasts, L = linear, Q = quadratic, and C = cubic effect of AFB_1_; ^3^ VFA = volatile fatty acids; ^4^ A: P = acetate to propionate ratio.

**Table 3:** Effect of incremental doses of aflatoxin B_1_ (AFB_1_) on nutrient digestibility of a lactating dairy cow diet in a dual-flow continuous culture system.

Digestibility ^3^, %	Treatment ^1^				SEM	*p*-Value ^2^		
	Control	AF50	AF100	AF150		L	Q	C
DM	55.0	54.5	55.4	56.0	0.73	0.38	0.92	0.19
OM	62.4	60.9	61.9	63.4	1.84	0.82	0.99	0.15
CP	59.7	59.1	60.7	61.0	2.35	0.57	0.94	0.59
NDF	59.1	57.5	54.8	55.3	2.42	0.12	0.51	0.97
ADF	41.6	37.2	42.2	43.2	2.85	0.57	0.57	0.20
Starch	93.3	92.7	92.8	93.3	0.35	0.48	0.91	0.12

^1^ Control = 0 μg/kg, AF50 = 50 μg/kg, AF100 = 100 μg/kg, and AF150 = 150 μg/kg; ^2^ contrasts, L = linear, Q = quadratic, and C = cubic effect of AFB_1_; ^3^ true digestibility for DM, OM, and CP; apparent digestibility for NDF, ADF, and starch.

**Table 4:** Effect of incremental doses of aflatoxin B_1_ (AFB_1_) on nutrient digestibility of a lactating dairy cow diet in a dual-flow continuous culture system.

Item	Treatment ^1^				SEM	*p*-Value ^2^		
	Control	AF50	AF100	AF150		L	Q	C
N flow, g/d								
Total N	2.30	2.32	2.34	2.38	0.07	0.41	0.58	0.56
NH_3_-N ^3^	0.43	0.41	0.39	0.45	0.05	0.62	0.52	0.35
NAN ^4^	1.87	1.90	1.95	1.93	0.09	0.32	0.88	0.98
Microbial N ^5^	1.06	1.07	1.11	1.10	0.04	0.20	0.95	0.82
Dietary N ^6^	1.27	1.27	1.23	1.25	0.07	0.55	0.78	0.85
Microbial efficiency ^7^	18.8	18.9	19.2	19.0	0.69	0.59	0.81	0.99
N efficiency ^8^	36.1	36.8	37.4	37.3	1.30	0.34	0.79	0.96

^1^ Control = 0 μg/kg, AF50 = 50 μg/kg, AF100 = 100 μg/kg, and AF150 = 150 μg/kg; ^2^ contrasts, L = linear, Q = quadratic, and C = cubic effect of AFB_1_; ^3^ NH_3_-N (grams/d) = mg/dL of effluent NH_3_-N × (grams of total effluent flow/100); ^4^ NAN = non-ammonia N. NAN flow (grams/d) = grams of effluent N − grams of effluent NH_3_-N; ^5^ microbial N flow (grams/d) = (NAN flow × atom percentage excess of ^15^N of effluent)/(atom percentage excess of ^15^N of bacteria); ^6^ dietary N flow (grams/d) = grams of effluent NAN − grams of effluent microbial N; ^7^ microbial efficiency = grams of microbial N flow/kilograms of OM truly digested; ^8^ N efficiency = (grams of microbial N/grams of available N) × 100.

## Data Availability

The data that support the findings of this study are available from the corresponding author upon reasonable request.

## Supplementary Materials

The following supporting information can be downloaded at: https://www.mdpi.com/article/10.3390/toxins15020090/s1, Figure S1. Effect of dose of aflatoxin B_1_ (AFB_1_) by sampling hour interaction on NH3-N concentration in dual-flow continuous culture system; Figure S2. Effect of dose of aflatoxin B_1_ (AFB_1_) by sampling hour interaction on pH in the dual-flow continuous culture system; Figure S3. Effect of dose of aflatoxin B_1_ (AFB_1_) by sampling hour on lactate concentration in the dual-flow continuous culture system; Figure S4. Effect of dose of aflatoxin B_1_ (AFB_1_) by sampling hour on total VFA concentration in the dual-flow continuous culture system; Figure S5. Effect of dose of aflatoxin B_1_ (AFB_1_) by sampling hour on acetate molar proportion in the dual-flow continuous culture system; Figure S6. Effect of dose of aflatoxin B_1_ (AFB_1_) by sampling hour on propionate molar proportion in the dual-flow continuous culture system; Figure S7. Effect of dose of aflatoxin B_1_ (AFB_1_) by sampling hour on iso-butyrate molar proportion in the dual-flow continuous culture system; Figure S8. Effect of dose of aflatoxin B_1_ (AFB_1_) by sampling hour on butyrate molar proportion in the dual-flow continuous culture system; Figure S9. Effect of dose of aflatoxin B_1_ (AFB_1_) by sampling hour on iso-valerate molar proportion in the dual-flow continuous culture system; Figure S10. Effect of dose of aflatoxin B_1_ (AFB_1_) by sampling hour on valerate molar proportion in the dual-flow continuous culture system; Figure S11. Effect of dose of aflatoxin B_1_ (AFB_1_) on hourly clearance rate of AFB_1_ in a dual-flow continuous culture system fed a lactating dairy cow diet.
