# Supplementary material for: The Effects of Incremental Doses of Aflatoxin B1 on In Vitro Ruminal Nutrient Digestibility and Fermentation Profile of a Lactating Dairy Cow Diet in a Dual-Flow Continuous Culture System
Source: Toxins (Basel). 2023 Jan 18;15(2):90. doi: 10.3390/toxins15020090 (PMC9964898; doi:10.3390/toxins15020090)
Supplement: Supplementary file 1 [file toxins-15-00090-s001.zip › toxins-2148081-supplementary.pdf]

SUPPLEMENTAL MATERIAL: The Effects of Incremental Doses of Aflatoxin B1 on In Vitro Ruminal Nutrient Digestibility and Fermentation Profile of a Lactating Dairy Cow Diet in a Dual-Flow Continuous Culture System

Felipe Xavier Amaro, Yun Jiang, Kathy Arriola, Matheus R. Pupo, Bruna C. Agustinho, Sarah L. Bennett, James R. Vinyard, Lais Tomaz, Richard R. Lobo, Andres Pech-Cervantes, Jose A. Arce-Cordero, Antonio P. Faciola, Adegbola Tolulope Adesogan and Diwakar Vyas

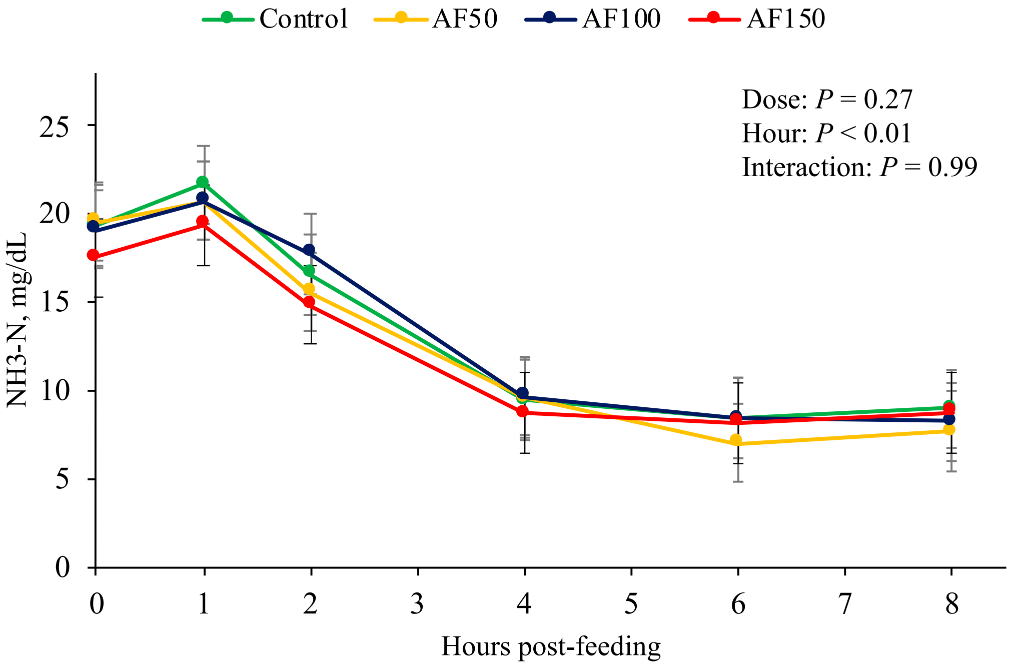

Figure S1. Effect of dose of aflatoxin B_1 (AFB_1) by sampling hour interaction on NH_3-N concentration in dual-flow continuous culture system.

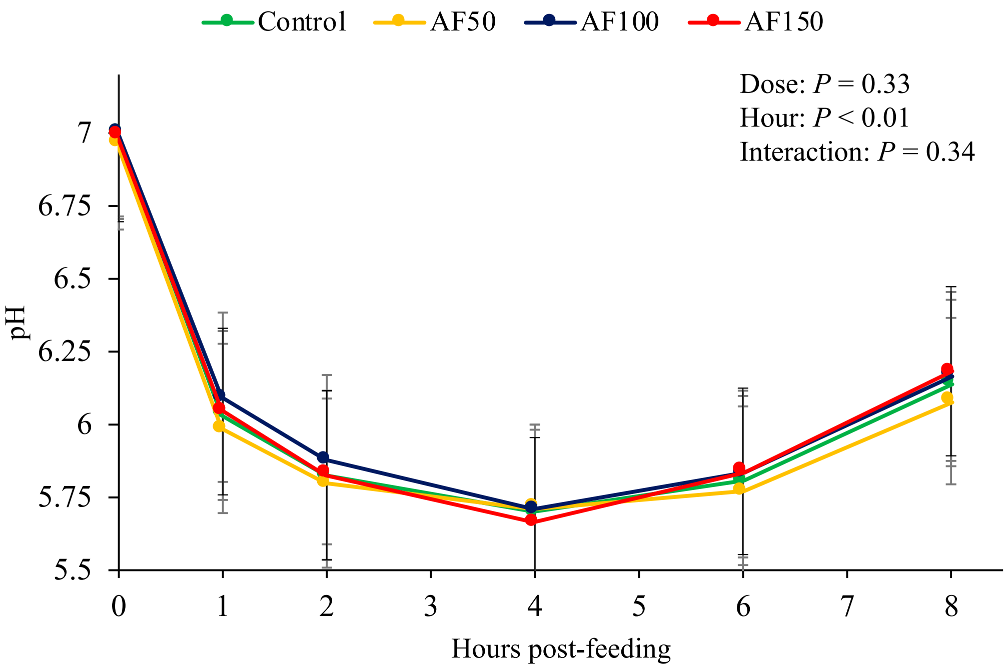

Figure S2. Effect of dose of aflatoxin B_1 (AFB_1) by sampling hour interaction on pH in the dual-flow continuous culture system.

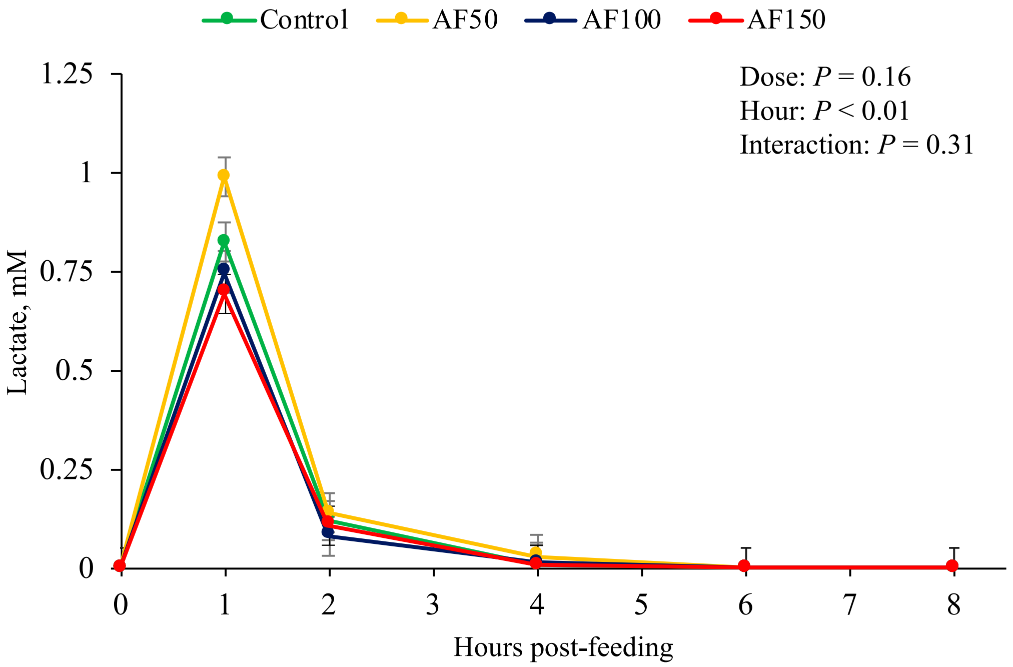

Figure S3. Effect of dose of aflatoxin B_1 (AFB_1) by sampling hour on lactate concentration in the dual-flow continuous culture system.

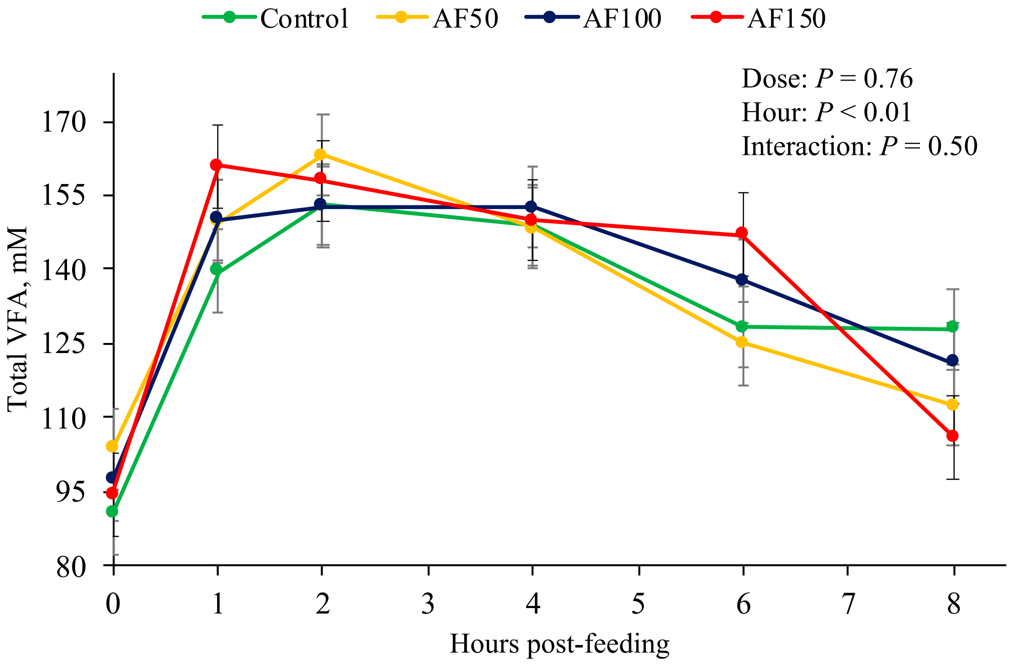

Figure S4. Effect of dose of aflatoxin B_1 (AFB_1) by sampling hour on total VFA concentration in the dual-flow continuous culture system.

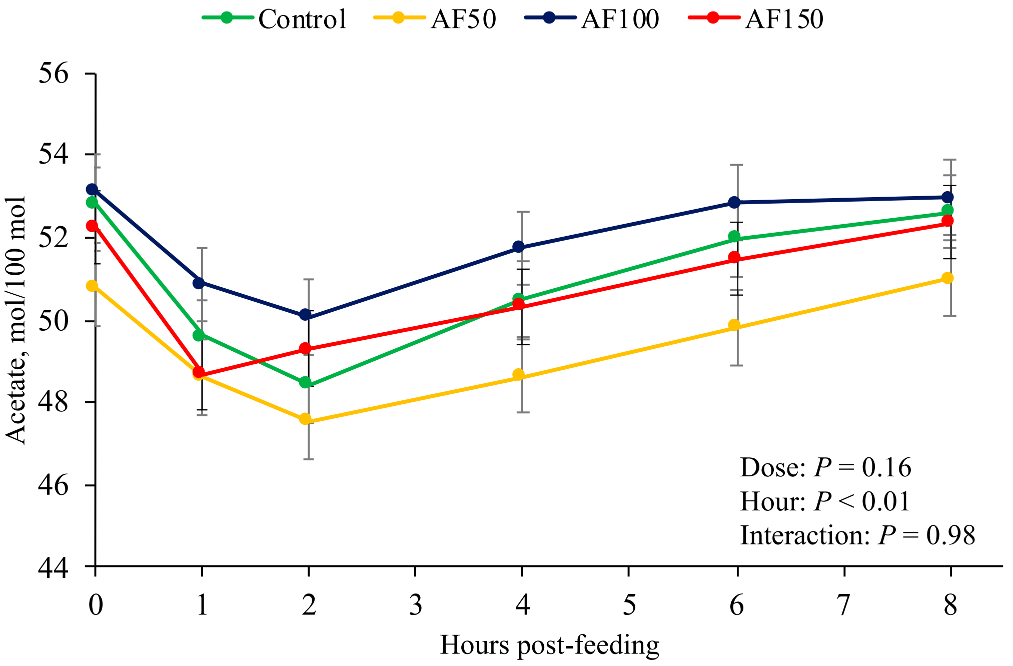

Figure S5. Effect of dose of aflatoxin B_1 (AFB_1) by sampling hour on acetate molar proportion in the dual-flow continuous culture system.

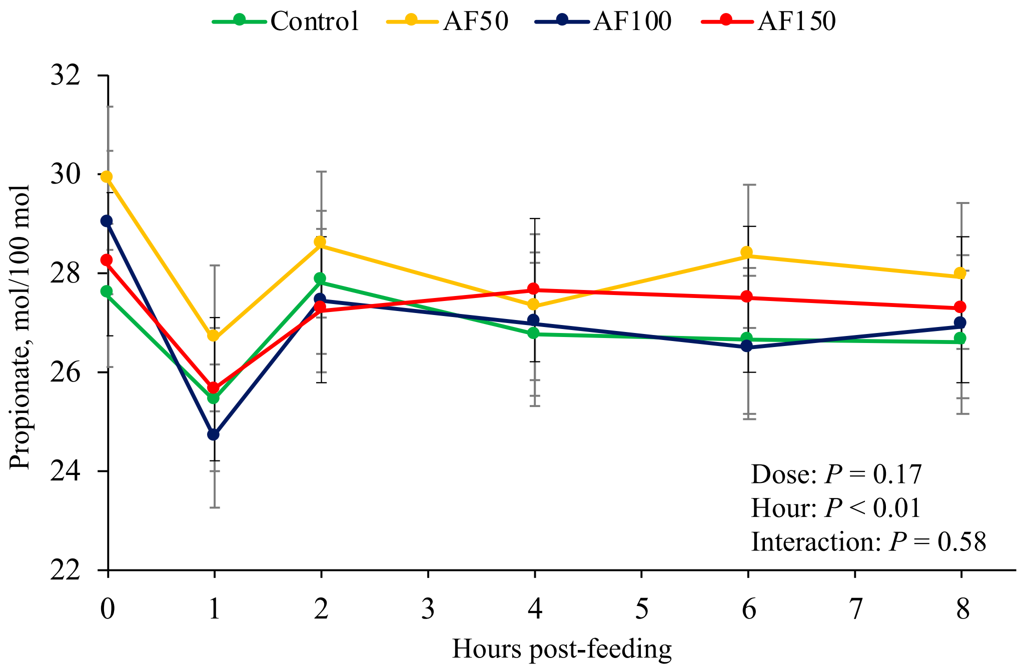

Figure S6. Effect of dose of aflatoxin B$_1$ (AFB$_1$) by sampling hour on propionate molar proportion in the dual-flow continuous culture system.

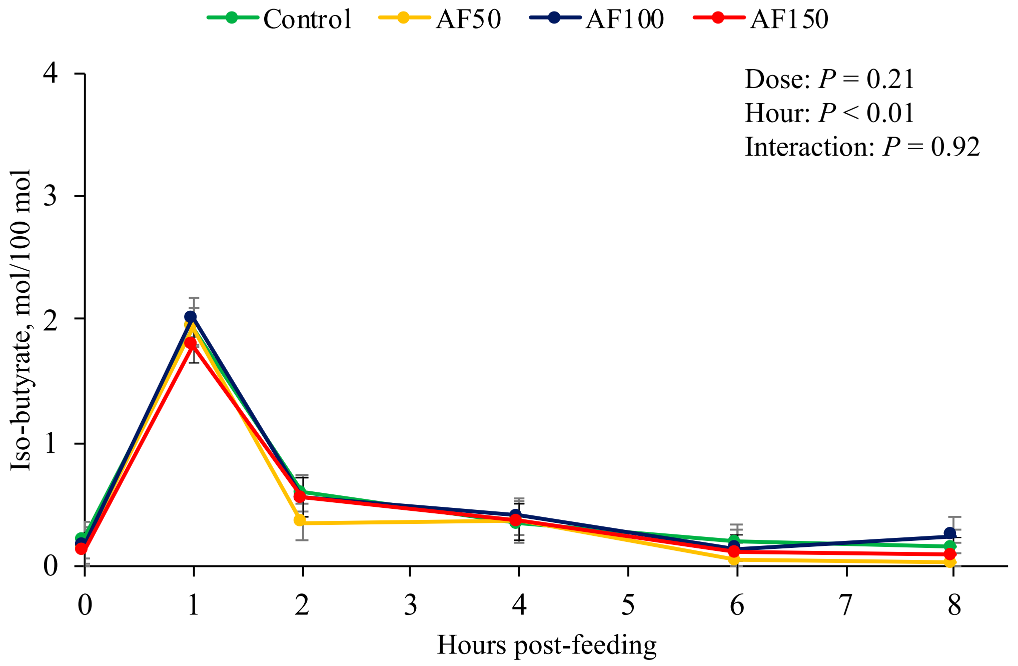

Figure S7. Effect of dose of aflatoxin B$_1$ (AFB$_1$) by sampling hour on iso-butyrate molar proportion in the dual-flow continuous culture system.

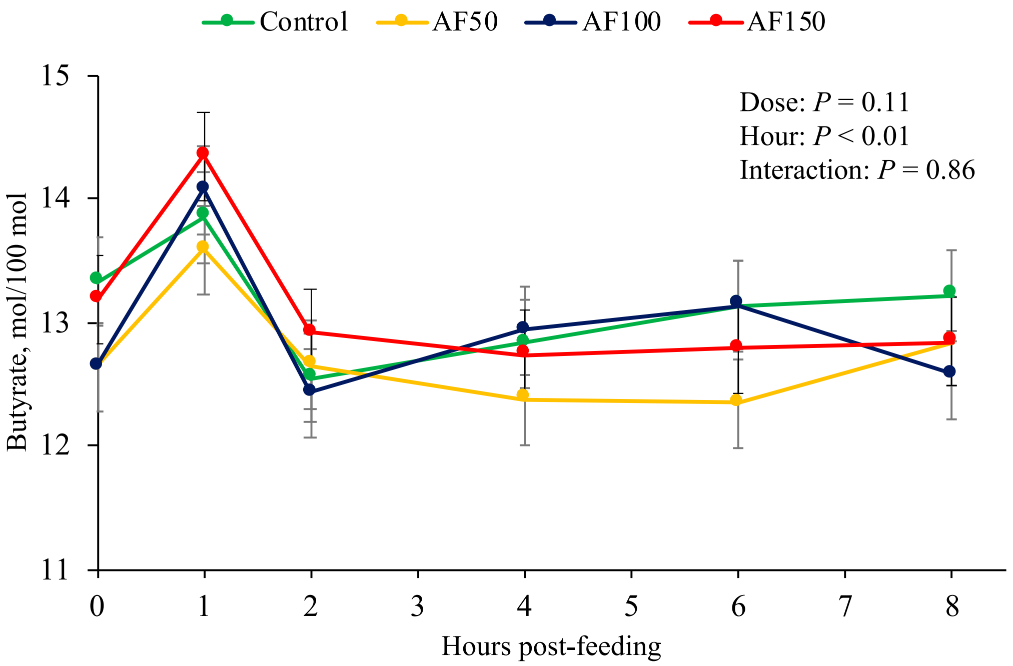

Figure S8. Effect of dose of aflatoxin B_1 (AFB_1) by sampling hour on butyrate molar proportion in the dual-flow continuous culture system.

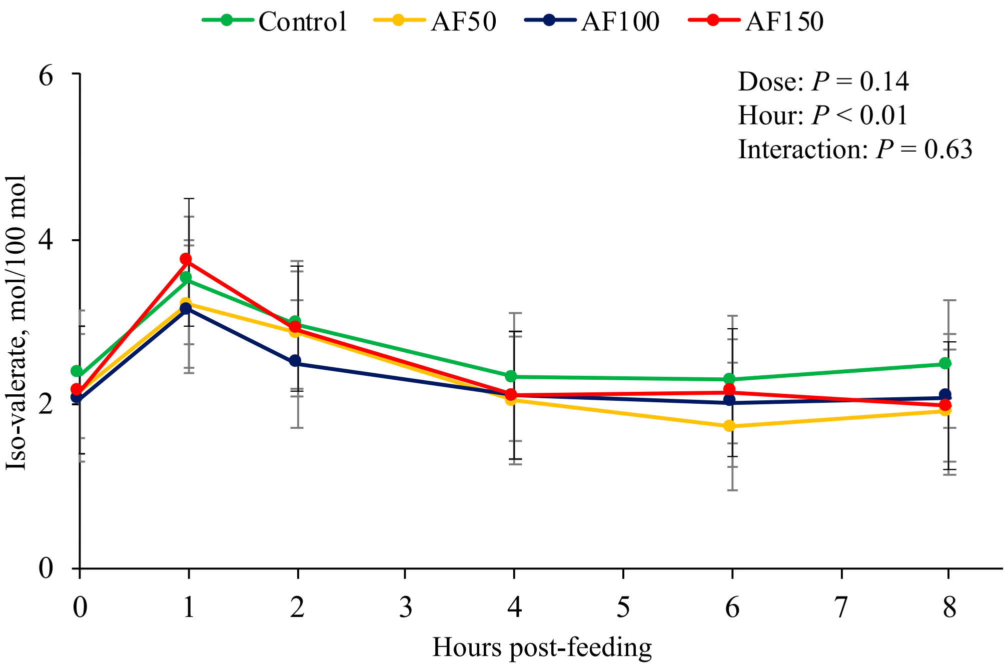

Figure S9. Effect of dose of aflatoxin B_1 (AFB_1) by sampling hour on iso-valerate molar proportion in the dual-flow continuous culture system.

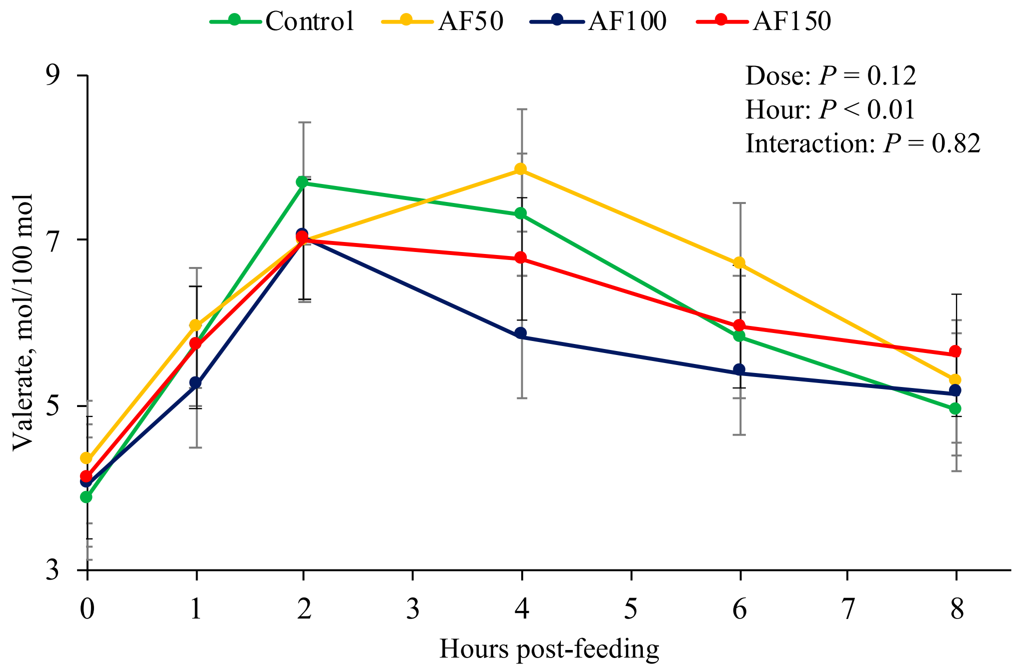

Figure S10. Effect of dose of aflatoxin B1 (AFB1) by sampling hour on valerate molar proportion in the dual-flow continuous culture system.

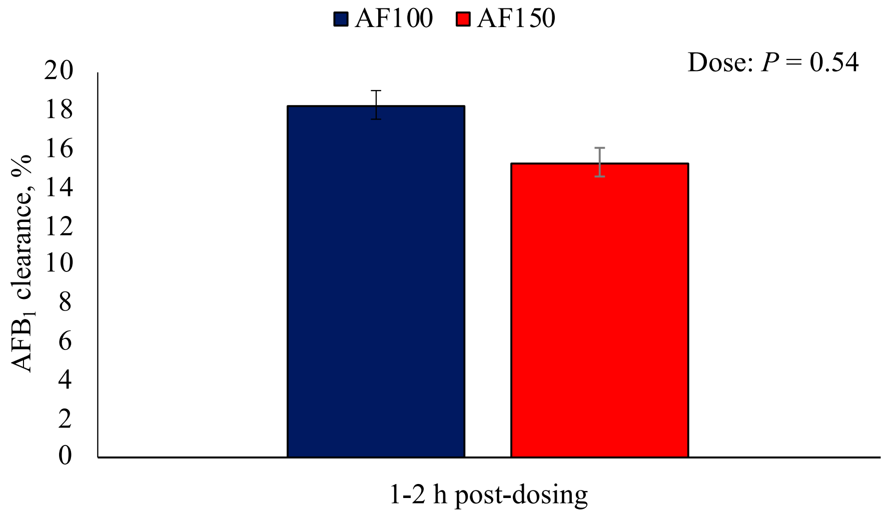

Figure S11. Effect of dose of aflatoxin B1 (AFB1) on hourly clearance rate of AFB1 in a dual-flow continuous culture system fed a lactating dairy cow diet.
